# Institutional Questions

**Published:** 1899-12-30

**Authors:** 


### INSTITUTIONAL QUESTIONS.

### Notification of Infectious Diseases.

"Sanitas" writes: What are the exact regulations governing the notification of cases of infectious disease by medical men called to attend such in private houses, and what are the diseases included under the term?

The following description is taken from the "Medical Directory": "In London districts, and in all other districts where the Notification Act has been adopted, every medical practitioner attending on or called in to visit a patient in any building, except an infectious hospital, or in any vessel, tent, or shed, must forthwith, on becoming aware that such patient is suffering from an infectious disease, send to the medical officer of health for the district a certificate stating the name of the patient, the situation of the building, and the infectious disease, and is liable to a penalty of 40s. in case of default. The local authority supply forms of certificate gratuitously, and pay to every practitioner for each certificate duly sent a fee of 2s. 6d. if the case occurs in his private practice, and 1s. if in his practice as medical officer of any public body or institution. Where there are two or more medical officers of health the certificate must be sent to the one in whose area the patient is. The term 'infectious disease' includes small-pox, cholera, diphtheria, membranous croup, erysipelas, scarletina, scarlet fever, typhus, typhoid, enteric, relapsing, continued, or puerperal fever; but the local authority may extend the definition temporarily or permanently."

